# How Do Growth and Sibling Competition Affect Telomere Dynamics in the First Month of Life of Long-Lived Seabird?

**DOI:** 10.1371/journal.pone.0167261

**Published:** 2016-11-30

**Authors:** Yuichi Mizutani, Yasuaki Niizuma, Ken Yoda

**Affiliations:** 1 Graduate School of Environmental Studies, Nagoya University, Chikusa-ku, Nagoya, Japan; 2 Faculty of Agriculture, Meijo University, Tempaku-ku, Nagoya, Japan; Estacion Experimental de Zonas Aridas, SPAIN

## Abstract

Telomeres are nucleotide sequences located at the ends of chromosomes that promote genome stability. Changes in telomere length (dynamics) are related to fitness or life expectancy, and telomere dynamics during the development phase are likely to be affected by growth and stress factors. Here, we examined telomere dynamics of black-tailed gull chicks (*Larus crassirostris*) in nests with and without siblings. We found that the initial telomere lengths of singletons at hatching were longer than those of siblings, indicating that singletons are higher-quality chicks than siblings in terms of telomere length. Other factors likely affecting individual quality (i.e., sex, laying date, laying order of eggs, and clutch size) were not related to telomere lengths. Within broods, initial telomere lengths were longer in older chicks than in younger chicks, suggesting that maternal effects, which vary with laying sequence, influence the initial lengths. Additionally, telomeres of chicks with a sibling showed more attrition between hatching and fledging than those of singleton chicks, suggesting that being raised with siblings can cause a sustained competitive environment that leads to telomere loss. High growth rates were associated with a low degree of telomere shortening observed in older siblings, perhaps because slower growth reflects higher food stress and/or higher aerobic metabolism from increased begging effort. Our results show that developmental telomere attrition was an inevitable consequence in two-chick nests in the pre- and post-hatching microenvironments due to the combination of social stress within the nest and maternal effects. The results of our study shed light on telomere dynamics in early life, which may represent an important physiological undercurrent of life-history traits.

## Introduction

Telomeres are nucleotide sequences located at the ends of chromosomes; they promote genome stability and shorten with each cycle of cell division in the absence of restoration [1]. In addition, telomeres are shortened by oxidative damage due to physiological and psychological stresses [2,3] and can be lengthened via the enzyme telomerase [4]. Telomere length appears to change most drastically during periods of growth and/or before sexual maturation when resources need to be allocated between different functions, as reported in several studies (e.g., in young *Homo sapiens* [5], medaka *Oryzias latipes* [6], European shag *Phalacrocorax aristotelis*, wandering albatross *Diomedea exulans* [7], and black-tailed gull *Larus crassirostris* [8]). For instance, in barnacle geese (*Branta leucopsis*), the rate of telomere shortening during a 2-year period in juveniles was greater than that in adults [9]. Because telomere length and change in telomere length (dynamics) are suggested to be related to fitness or life-expectancy in animals [10–13], telomere dynamics in early life can potentially affect survival and fitness similarly to, or to a greater degree, than at adulthood. For example, Western jackdaw (*Corvus monedula*) nestlings that experienced greater telomere attrition during the growth period had lower survival rate in later life [14]. Thus, it is important to examine how telomeres of young animals change throughout the growth period in order to understand the evolution of life-history traits such as lifespan and growth.

The factors governing telomere dynamics in the developmental phase of young individuals are as follows: increased cell division [7,15], high telomerase activity [16], and enhanced oxidative stress levels [17–19]. These three mechanisms probably interact and their effects on telomere length in young animals need to be carefully dissected [7,8,20].

Several ecological factors affecting telomere dynamics during the early period of life via these causal mechanisms are considered important. First, elevated growth rates lead to increased aerobic metabolism, which leads to the production of reactive oxygen species and oxidative stress [21,22]. For instance, Geiger *et al*. [23] reported that excessive catch-up or compensatory growth induced higher oxidative damage and extensive telomere shortening in later-born chicks of king penguins (*Aptenodytes patagonicus*). Alternatively, slower growth may be related to a high level of food stress and/or a lower level of telomerase activity [24], which can produce the opposite effect [25].

Secondly, sibling competition, which is often observed in birds [26,27] and mammals [28], may increase the metabolic rate of the young, which in turn, can affect telomere dynamics. Intense begging behavior in yellow-legged gull chicks (*Larus michahellis*) was associated with lower nutritional condition and higher oxidative stress [29]. Therefore, additional siblings are expected to decrease nutrition per chick and increase the cost of begging for food. This process may be exacerbated by hatching asynchrony, which creates competitive asymmetry within broods [30]. In the European starling (*Sturnus vulgaris*), experimental manipulation of nestling competitive hierarchies indicated that more advantaged siblings experienced less telomere shortening than disadvantaged nestlings [31,32]. Similarly, in free-living jackdaws, increased brood size led to decreased fledging mass and telomere length compared to brood-size reduction [14]. Previous studies examined species that naturally have large brood sizes (e.g., 4–5 in jackdaw [14] and starling [31,32]) in which the hierarchy in a nest is changed or the brood size is reduced to two or more. However, it is difficult to manipulate and quantify the level of competition among chicks in brood manipulation experiments, because social interaction is complicated in large families and the relationship between brood size and competition is not straightforward [33–36]. To dissect the effect of growth and sibling competition, physiological comparisons between chicks with zero competition (i.e., singleton chicks) and chicks with competition between siblings, while taking differences in growth rate into account, may shed light on the effects of competition on telomere dynamics.

In this study, we investigated how growth and competition are related to telomere dynamics during the nestling period of a long-lived seabird, the black-tailed gull, by comparing telomere lengths among chicks with different growth rates and brood sizes (i.e., naturally occurring broods of one or two; [37]). We also considered other factors that are likely to affect individual quality (i.e., sex, laying date, laying order of eggs, and clutch size). We examined the following hypotheses: (1) whether telomere lengths are different at hatching and at fledging between singleton chicks and chicks with a sibling; (2) whether chicks with a higher growth rate show more telomere shortening or lengthening; and (3) whether sibling rivalry promotes telomere shortening in two-chick nests during the first month of life.

## Materials and Methods

### Ethics statement

The procedures used in this study adhered to the guidelines of the Animal Experimental Committee of Nagoya University. The protocols followed for the survey and to capture chicks at Kabushima Island, a national natural monument, were approved by the Agency for Cultural Affairs, Japan (permit number 21-4-1047) and the Aomori Prefectural Government (permit number 4–2010).

### Fieldwork

Fieldwork was conducted from the middle of April to July 2010 during the chick-rearing period of the black-tailed gull following an incubation period of about 25 days [37] on Kabushima Island, Japan (40°32′18 N, 141°33′27 E). We recorded the number of eggs, laying date, hatching order, and number of chicks in the nest, and checked the nests every day from pre-laying to hatching. We confirmed that the hatching order was the same as the laying order. Chicks were individually marked with colored plastic rings on their leg.

We measured the body mass (BM), bill depth, head length, tarsus length, and natural wing length, and sampled blood from 41 chicks from 27 nests. There were 13 singletons and 14 nests containing two chicks. Younger siblings hatched 0–3 days (median 1 day) after the older sibling hatched. The original clutch sizes were not different between one-chick and two-chick nests (*t* = 0.065, *p* = 0.95, *t*-test). In one-chick nests, the clutch size varied from one (*n* = 1) to three (*n* = 3), but most nests (*n* = 9) contained two eggs. The singletons hatched from the first (*n* = 8), the second (*n* = 4), and the third (*n* = 1) eggs. In the two-chick nests, the clutch size was two, except for two nests (clutch size of three), and older chicks hatched from the first eggs and the younger chicks hatched from the second eggs.

We checked each nest every 5 days following hatching of the singleton or the younger sibling and measured BM and external measurements of all chicks in the nest. We used BM and external measurements taken at the closest age point for older siblings. We collected blood from the chicks at 1 and 30 days of age. Each nest was enclosed within fences sufficiently large for breeding territories to prevent any interference from neighbors, who sometimes intrude and attack chicks [38]. We analyzed data from nests in which brood size did not change from hatching to fledging. Therefore, the fledge size (the number of chicks fledged) was equal to the brood size.

### DNA sampling and telomere measurements

Blood samples (15–30 μl) were collected by puncturing the brachial vein when chicks were 1- and 30-days old. Immediately after collection blood samples were stored in a stock solution prepared by mixing 180 μl of PBS with 20 μl Proteinase K (Qiagen) at room temperature, with no direct sunlight until laboratory analysis. Genomic DNA was extracted from whole-blood samples using a DNeasy Blood and Tissue Kit (Qiagen) and terminal restriction fragments were analyzed by Southern blotting and DNA hybridization with non-radioactive DNA probes following the method described by Mizutani *et al*. [39]. We analyzed telomere length using TELOMETRIC (ver. 1.2 [40]). We electrophoresed the same individual samples (i.e., 1-day and 30-days old chicks) in the same gel to avoid differences in color distorting the measurements of within-individual changes.

Chromosomal distributions of telomeric repeats vary according to bird species [41]. If telomeric sequences are found in the pericentric regions of chromosomes (interstitial telomeres), it could lead to inaccurate measurements of telomere length. Before measuring telomere length, we confirmed that black-tailed gulls did not have interstitial telomeric sequences on chromosomes using fluorescence *in situ* hybridization in cultured fibroblasts (Y. Mizutani, unpublished data). Therefore, it was not necessary to consider the effect of interstitial telomeric sequences in this study. Sex was determined using a PCR-based method [42].

### Statistical analysis

We defined the telomere length of 1-day-old chicks as the initial telomere length (ITL), and telomere length of 30-day-old chicks as the final telomere length (FTL). Change in telomere length (CTL) was defined as the FTL minus the ITL, with more negative values indicating a higher degree of telomere shortening during the nestling period. All statistical analyses were conducted using R version 3.2.4 [43]. Mean values are presented with ± 1 standard deviation (SD).

We used linear mixed models fitted by maximum likelihood estimations using the R package lme4 [44] to examine which factors affected ITL and CTL. The dependent variables were normally distributed (ITL: *p* = 0.51; CTL: *p* = 0.12, Kolmogorov–Smirnov tests). Nest identity was set as a random factor in the linear mixed models. We selected the most parsimonious model among all possible candidate models based on Akaike’s information criterion corrected for small sample size (AICc). The best model is the one that minimizes the value of AICc. Residual plots and normal probability plots were used to visually check for deviations from normality among residuals in the best model. We calculated marginal (*R*^*2*^_*m*_) and conditional *R*^*2*^ (*R*^*2*^_*c*_) values to evaluate the goodness of fit for the best model [45].

First, we examined which factors affected the ITL of chicks. We entered sex, laying date, laying order nested in clutch size, and sibling order nested in brood size, and their interactions, as independent variables, possible parameters affecting individual quality at hatching, in the full model. Secondly, we examined whether CTL was dependent on sex, “mass gain”, “size gain”, sibling order nested in brood size, and their interactions. We distinguished statural growth (temporal variation in body size) and mass gain (temporal variation in body mass) [46,47]. The mass gain was defined as the BM at 20 days of age minus the BM at 5 days of age, during which the BM increased linearly from approximately 100 to 500 g [37,48]. We conducted a principal components analysis (PCA) to obtain a composite body-size index for chicks by incorporating four measurements taken at hatching and fledging: bill depth, head length, tarsus length, and natural wing length. We did not include BM in the PCA of individuals, because BM varies with the timing of feedings, which occur at random during the day. We used the first PC (PC1) score as body size in the analysis. The PC1 score explained 58.0% and 67.4% of the variation in the measurements at hatching and fledging, respectively. We calculated the PC1 scores of size gain, in which measurements from 5- and 20-day-old chicks were pooled. The PC1 score explained 97.1% of the variation. The body-size gain was defined as the body size at 20 days of age minus that at 5 days of age. We compared BM and body size at hatching and fledging among singletons, older siblings, and younger siblings using ANOVAs. These variables were normally distributed (BM at hatching: *p* = 0.97; BM at fledging: *p* = 0.79, body size at hatching: *p* = 0.96; body size at fledging: *p* = 0.90, Kolmogorov–Smirnov tests).

## Results

**Fig 1:**
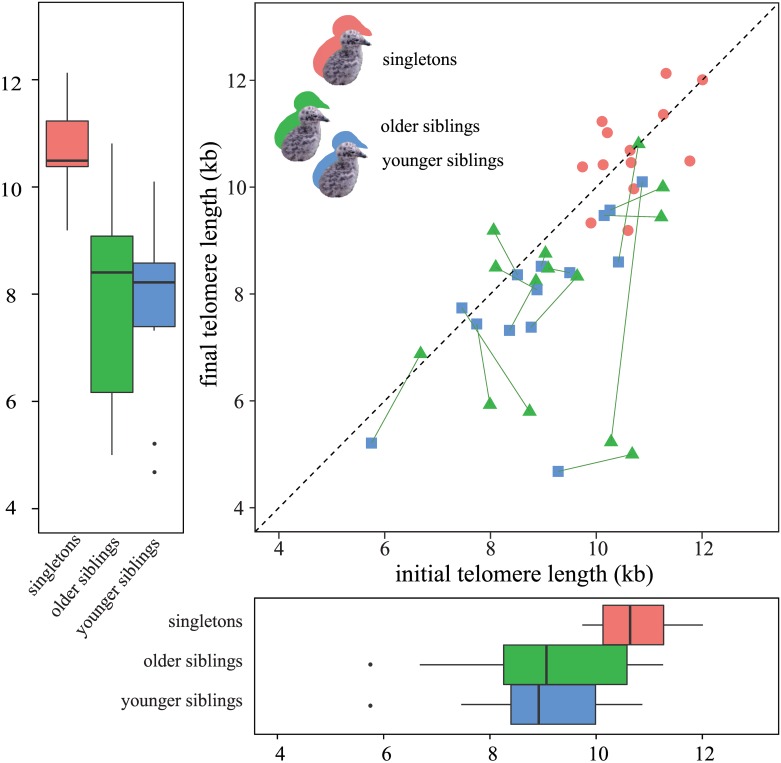
Telomere length of chicks at hatching and fledging. The red circles and boxes represent single chicks. The green triangles and boxes and blue squares and boxes represent older and younger siblings, respectively. In the scatter plot, the line between a triangle and a square connects siblings from the same nest. The dotted line represents equality of the telomere lengths: in which chicks over the line experienced telomere lengthening from hatching to fledging, whereas chicks below the line experienced telomere attrition. The siblings had shorter telomere length at hatching and fledging and experienced more drastic telomere attrition than singletons during the nestling period.

We obtained telomere lengths from 41 chicks (16 males and 25 females). ITL for all chicks was 9.62 ± 1.40 kb (singletons: 10.70 ± 0.71 kb, older siblings: 9.32 ± 1.39 kb, younger siblings: 8.92 ± 1.36 kb; Fig 1). FTL for all chicks was 8.78 ± 1.94 kb (singletons: 10.67 ± 0.89 kb, older siblings: 7.90 ± 1.83 kb, younger siblings: 7.92 ± 1.52 kb; Fig 1). CTL for all chicks was –0.84 ± 1.50 kb (singletons: –0.03 ± 0.80 kb, older siblings: –1.42 ± 1.99 kb, younger siblings: –1.00 ± 1.16 kb).

**Fig 2:**
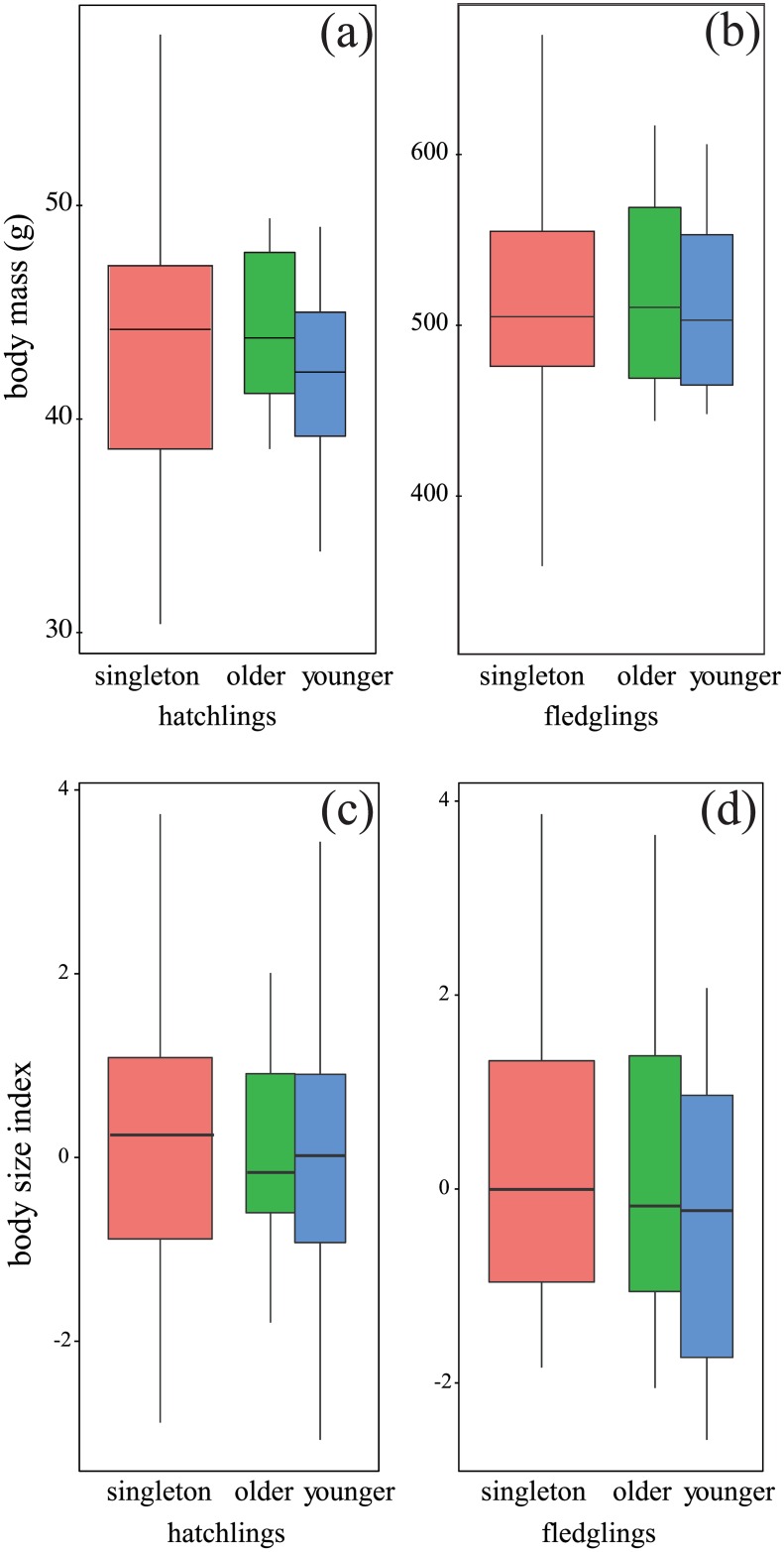
Body mass and body size index (PC1 score). Body mass at hatching (a) and fledging (b), body size index at hatching (c) and fledging (d). The red boxes represent singleton chicks, and the green and blue boxes represent siblings: the left corresponds to older chicks and the right corresponds to younger chicks.

Hatching BM for all chicks was 43.20 ± 5.11 g (singletons: 43.5 ± 7.03 g; older siblings: 44.3 ± 3.62 g; younger siblings: 41.8 ± 4.21 g; Fig 2a). Fledging BM for all chicks was 511.15 ± 68.71 g (singletons: 514.9 ± 79.04 g; older siblings: 518.5 ± 57.46 g; younger siblings: 500.3 ± 72.50 g; Fig 2b). PC1 scores at hatching in singletons, older siblings, and younger siblings were 0.09 ± 1.76, 0.08 ± 1.11, and –0.17 ± 1.73, respectively (Fig 2c), whereas those at fledging in singletons, older siblings, and younger siblings were 0.21 ± 1.72, 0.20 ± 1.72, and –0.40 ± 1.54, respectively (Fig 2d). BM and body size at hatching and fledging showed no significant difference among the three groups (BM at hatching: *F*_*2*, *38*_ = 0.92, *p* = 0.41; BM at fledging: *F*_*2*, *38*_ = 0.26, *p* = 0.77, body size at hatching: *F*_*2*, *38*_ = 0.13, *p* = 0.88; body size at fledging: *F*_*2*, *38*_ = 0.60, *p* = 0.56; Fig 2).

For ITL, the best model included brood size and the interaction between brood size and sibling order (Table 1) with *R*^*2*^_*m*_ of 0.327 and *R*^*2*^_*c*_ of 0.867. The best model indicated that singletons had a longer ITL than siblings, and younger siblings had a shorter ITL than older sibling (Table 1).

**Table 1:** Linear mixed-effects model estimates of initial telomere length (ITL) in relation to brood size and sibling order.

Fixed effects	Estimate	*t*	*p*
(Intercept)	10.7 ± 0.30	35.33	< 0.0001
Brood size	–1.38 ± 0.42	–3.28	0.0025
Brood size: Sibling order*	–0.39 ± 0.18	–2.15	0.0473

* Relative to older siblings

**Fig 3:**
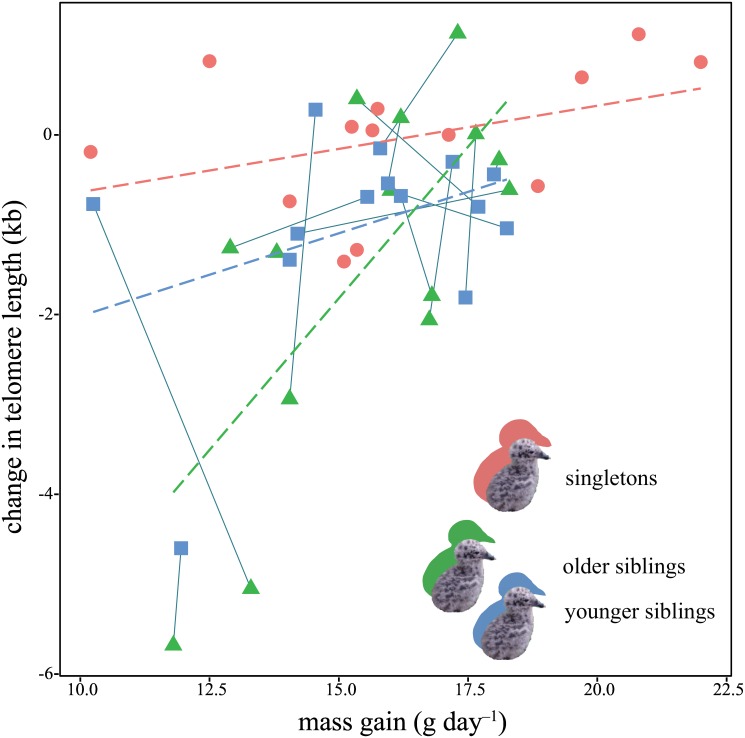
Relationship between mass gain and change in telomere length during the nestling period. The red circles represent singleton chicks. The green triangles and blue squares represent older and younger siblings, respectively. The solid lines connect siblings. Dashed lines represent regression lines for singletons (red), older siblings (green), and younger siblings (blue), respectively.

For CTL, the best model included mass gain, brood size, and interactions among brood size, mass gain, and sibling order (Table 2) with *R*^*2*^_*m*_ of 0.474 and *R*^*2*^_*c*_ of 0.534. The best model indicated that the CTL was larger in singletons than in siblings. This model did not show a significant relationship between the mass gain and CTL for all chicks, but the slope was steeper for siblings than for singletons (Table 2; Fig 3). Moreover, the CTL of younger siblings was larger than that of older siblings and the slope of the CTL on the mass gain was steeper for older siblings than for younger siblings (Table 2; Fig 3).

**Table 2:** Linear mixed-effects model estimates of change in telomere length (CTL) during the nestling period in relation to mass gain, brood size, and sibling order.

Fixed effects	Estimate	*t*	*p*
(Intercept)	–1.60 ± 1.55	–1.03	0.3075
Brood size	–10.11 ± 2.71	–3.73	0.0006
Mass gain	0.10 ± 0.09	1.03	0.3071
Brood size: Mass gain	0.56 ± 0.17	3.33	0.0018
Brood size: Sibling order*	8.35 ± 2.87	2.91	0.0076
Brood size: Mass gain: Sibling order*	–0.51 ± 0.18	–2.78	0.0104

* Relative to older siblings

## Discussion

In this study, we found that singleton had already longer telomeres at hatching than chicks from two-chick broods. Singletons were therefore considered chicks of relatively high quality than siblings in terms of telomere length, although ITL was not related to factors that are likely to affect individual quality, i.e., sex, laying date, laying order of eggs, and clutch size. Assuming that telomere length is partly heritable [49] (maternally inherited in birds [50,51]), a large ITL was expected in dead chicks hatched in nests where only a single chick survived. However, the mortality rate in one-chick nests was higher (53.6%) than that in two-chick nests (6.7%); therefore, the cause of egg death may not be related to telomere length.

The high mortality rate observed in one-chick nests could lead to the expectation that inexperienced young parents rear singletons and have long telomeres. However, young birds do not necessarily have a small brood size (Y. Mizutani, unpublished data) nor long telomeres [8,39]. Furthermore, in European shag, the parental age ranged from 2 to 22 years and did not influence the ITL of the chicks [52].

Alternatively, singleton chicks (eggs) might lose competition with siblings following the death of its/their sibling(s) at some point during the incubation period. Eggs in a nest may communicate and compete with each other using vibrations or volatile chemicals [53]. These egg-egg interactions may accelerate decrease of ITL in siblings rather than in singletons under non-competition conditions. Another hypothesis is that mothers may transfer more antioxidants in eggs of singleton broods than in eggs of sibling broods, which results in higher antioxidant defenses and longer telomeres in singletons than in siblings. Such differential antioxidant allocation between eggs has already been described in several bird species [54–57].

Younger siblings exhibited shorter telomere length at hatching than older siblings. Female parents increase the testosterone concentration or adjust other components of later-laid eggs to mitigate or enhance sibling competition after hatching (i.e., maternal effect [58]), which may also affect telomere length. We showed that the telomere length of younger siblings at hatching tended to be shorter than that of the older sibling. In this species, the testosterone concentration in yolk increases with laying order [59], which has also been reported in several other gull species [56,60,61]. Early exposure to steroid hormones induces oxidative stress [62] and accelerates telomere attrition in embryos [63], implying that maternal hormones in eggs have negative effects on the ITL of offspring.

Telomeres of chicks with siblings showed more attrition between hatching and fledging than those of singleton chicks did, suggesting that interactions between siblings, probably of a competitive nature, affected telomere dynamics. Being raised with siblings can cause a sustained competitive environment, which elevates aerobic metabolism and stress hormone levels (corticosterone) to a higher level in siblings than in singletons. This is observed in the common tern *Sterna hirundo* [64], although parents of black-tailed gulls seem to be able to provide enough care to one- and two-chick-broods, but not to three-chick-broods in unfavorable environmental conditions [37].

Although it was difficult to determine possible sources of competition in this study, siblings from two-chick broods may compete for food because the relationship between CTL and mass gain changed between singletons and siblings, and even between older and younger siblings. The singletons retained their telomere length during the growth period, whereas older siblings were more likely to decrease their CTL and show a more positive relationship between mass gain and CTL than younger siblings. The relatively unchanged telomere length of fast-growing older chicks was consistent with the yellow-legged gull chicks [25], indicating that faster growth may be related to a lower level of food-related stress or vulnerability. One explanation could be that the fast-growing chicks might get more antioxidant from dietary sources (e.g., vitamins C and E [22,56,57,65]) than slow-growing chicks.

In addition, physical effort and stress might affect telomere dynamics during the growth period, as observed in nestling European shags exposed to different degrees of stress (unhandled chicks, chicks handled and given fish oil, and chicks handled and given corticosterone), with increased telomere shortening observed in stressed groups compared with the control group [66]. If siblings engage in physical competition, differences in body size might be important for determining stress, which may result in different degrees of telomere shortening in chicks of different sizes. However, earlier-born siblings had physical advantages in body size, but decreased their telomere length more than later-born siblings. This contrasted with the finding of Nettle *et al*. [31,32] who found that disadvantaged chicks within a nest experienced more telomere attrition than advantaged nestlings. In our studied species, nonphysical contests such as scramble begging may cause oxidative damage to siblings [29], because siblings often engaged in vigorous begging with siblings when their parents returned with food items. Younger siblings could try to compensate for their relative size-disadvantage, although the body size at birth was similar to that of older siblings, by increasing their begging effort, which can cause telomere shortening. Nonetheless, we observed the opposite trend, such that older siblings experienced more attrition of telomere length than younger siblings did. The level of begging by individuals may not simply increase with brood size [67], and different begging strategies may be employed within broods [68]. These unobserved behavioral processes might result in a different relationship between mass gain and CTL in chicks of this species.

### Implications

The findings of this study suggest that parents end their reproduction for the season with either large numbers of offspring with short-telomeres or small numbers of offspring with long-telomeres. Telomere length during early development has been shown to be a strong predictor of lifespan [69]. Therefore, telomeres potentially constrain brood size. Assuming that telomere lengths of siblings remain 20% shorter than those of singletons until the age of first reproduction (approximately age 4; early-life telomere handicap cannot be overcome over a year [70]), telomere length of singletons at age 4 would be 14 kb with a 0.3-kb decrease per year during adulthood. Assuming that the lowest critical length of the telomere is 9 kb (because no adults have telomeres <9 kb in length derived from 25 adult black-tailed gulls [39]), then the life expectancy of singletons and siblings would be approximately 21 and 11, respectively, although this is an estimate because telomere attrition of adults is affected by many environmental factors [39,71,72]. If the fitness of rearing singletons and siblings is equal, natural selection would favor both brood sizes and generate variation in brood size in the population. As several studies have already shown that the brood size has long-lasting effects on metabolic rate in later life [73], reproductive output [74], and longevity [75], future studies should explore the potential role of telomere dynamics for clutch and/or brood size determination, which may serve as a novel physiological basis of life-history trade-offs [4,17,21,76], but see [77] and may affect offspring adult phenotypes [78].
